# Measuring Asymmetry in Time-Stamped Phylogenies

**DOI:** 10.1371/journal.pcbi.1004312

**Published:** 2015-07-06

**Authors:** Bethany L. Dearlove, Simon D. W. Frost

**Affiliations:** Department of Veterinary Medicine, University of Cambridge, Cambridge, United Kingdom; University of California San Diego, UNITED STATES

## Abstract

Previous work has shown that asymmetry in viral phylogenies may be indicative of heterogeneity in transmission, for example due to acute HIV infection or the presence of ‘core groups’ with higher contact rates. Hence, evidence of asymmetry may provide clues to underlying population structure, even when direct information on, for example, stage of infection or contact rates, are missing. However, current tests of phylogenetic asymmetry (a) suffer from false positives when the tips of the phylogeny are sampled at different times and (b) only test for global asymmetry, and hence suffer from false negatives when asymmetry is localised to part of a phylogeny. We present a simple permutation-based approach for testing for asymmetry in a phylogeny, where we compare the observed phylogeny with random phylogenies with the same sampling and coalescence times, to reduce the false positive rate. We also demonstrate how profiles of measures of asymmetry calculated over a range of evolutionary times in the phylogeny can be used to identify local asymmetry. In combination with different metrics of asymmetry, this combined approach offers detailed insights of how phylogenies reconstructed from real viral datasets may deviate from the simplistic assumptions of commonly used coalescent and birth-death process models.

## Author Summary

Phylogenetic trees of viruses sampled from different individuals provide clues to the dynamics of transmission. The extent to which the tree is asymmetric may be influenced by biological factors such as differences in infectiousness or contact rates between individuals, but also by nuisance factors such as the pattern of sampling. We have devised a simple statistical test for asymmetry, which controls for sampling patterns and potentially complex temporal dynamics by conditioning on the sampling and coalescence times in a phylogeny, and can also detect whether specific clades in the phylogeny drive patterns of asymmetry. We apply our approach to data on HIV, influenza A virus H5N1, and ebola virus.

## Introduction

Genetic approaches to investigating infectious diseases are well-established, exploiting the naturally high genetic diversity in pathogen populations such as HIV and influenza to reconstruct both their evolutionary and epidemiological dynamics [1]. Phylogenies contain potentially large amounts of information on disease dynamics, and can help reveal the disease incidence and prevalence, changes in historical population size, and population substructure [2–4]. However, there can be confounding factors when trying to convert evolutionary dynamics into epidemiological quantities such as transmission rates, and ideally we want to be able to explicitly model viral transmission in an evolutionary framework, taking into account features such as the host population structure (for example, differences in contact rates between groups of individuals) and the natural course of infection (for example, differences in infectiousness during the acute and chronic phases of HIV infection) [5].

One way to investigate the extra biological complexity of such patterns is to consider the shape or branching structure of the phylogeny, a feature that is arguably underused despite being relatively straightforward to infer. Evidence of asymmetry in a tree reflects heterogeneity in the population that has arisen due to the processes by which a tree has grown [6]; previous work suggests that evidence of asymmetry in a phylogenetic tree can arise due to selection [2], heterogeneity in contact rates [7] and population structure [5]. Since many tree models assume homogeneity in the population, it is important to be able to identify which parts of the tree might be driving asymmetry, and whether or not this is problematic under the modelling assumptions—preferably before running computationally expensive analyses.

It is common to analyse viral datasets sampled over multiple timepoints. As viruses, including RNA and ssDNA viruses, evolve rapidly, phylogenetic reconstruction gives rise to trees with root-to-tip distances that reflect, in part, sampling times. However, such trees are more likely to be asymmetric, resulting in standard metrics developed for homochronous sampling being implicitly biased (see Supplementary Information of Frost and Volz (2013) [5]). This is due to the fact that most metrics use the topological distance (that is, the number of nodes traversed between two points in the tree), and isolates sampled earlier in the history of the phylogeny will tend to have fewer nodes between them and the root of the tree.

In this paper, we propose a permutation-based approach that allows an observed phylogeny to be compared to random phylogenies with the same sampling and coalescence times. This approach can also be used to assess asymmetry throughout evolutionary history in a rooted tree, therefore also allowing areas of local asymmetry to be identified in addition to a single global value at the root of the tree. We demonstrate this approach on three datasets with different expected types of heterogeneity, illustrating the imprint of various transmission dynamics on viral phylogenies.

## Materials and Methods

### Measuring phylogenetic tree shape

There are a number of ways to measure the balance of a phylogeny. Most approaches consider either the topological distance (the number of nodes) between two parts of the tree, for example Sackin’s index, or the balance of each internal node by comparing the number of leaves in the left and right subtrees below it, for example Colless’ index [8–11]. Here, we consider two measures of asymmetry: Sackin’s index [11], and the number of cherries [12], although the methodology can easily be extended to other metrics.

Sackin’s index is the total topological distance between the leaves and root of the tree. If *d*_*j*_ is the number of nodes to be traversed between each leaf *j* and the root, then Sackin’s index is the total over all leaves,

$$\begin{array}{c} I_{S}=\sum_{j}d_{j}. \end{array}$$ (1)

In trees where the tips have been sampled at the same time, the expected Sackin’s index, 𝔼(*I*_*S*_(*n*)), for *n* isolates in the sample is given by:

$$\begin{array}{c} 𝔼\left(I_{S}\left(n\right)\right)=2n\sum_{k=2}^{n}\frac{1}{k} \end{array}$$ (2)

under the Yule or coalescent models [13]. For large *n*, 𝔼(*I*_*S*_(*n*)) ≈ 2*n* log(*n*). Since the expected value of the Sackin’s index increases with the tree sample size, it is common to either divide the statistic by *n* (i.e. the mean topological distance from root to tip), or use the following standardisation proposed by Leventhal et al. [7]:

$$\begin{array}{c} {\bar{I}}_{S}\left(n\right)=\frac{I_{S}\left(n\right)-𝔼\left(I_{S}\left(n\right)\right)}{𝔼(I_{S}\left(n\right))}. \end{array}$$ (3)

However, since the permutation method outlined in this paper compares an observed tree to those of the same size (i.e. like with like), we simply use the non-standardised version here. We use the function sackin.test in the apTreeshape R package to test the hypothesis of asymmetry in the tree, comparing the observed value to 10,000 trees simulated under the Yule model [14, 15].

A cherry is formed when two tips share a direct ancestor. In an asymmetric tree, tips generally coalesce with branches earlier in the ancestry of the tree, and therefore fewer cherries are expected than with a balanced tree. Under the Yule or coalescent model, the expected number of cherries, *C*_*n*_, in a tree with *n* taxa is *n*/3, and for a uniform tree is *n*/4 [12]. In addition, McKenzie and Steel showed that the number of cherries is asymptotically normal with

$$\begin{array}{c} \frac{C_{n}-n/3}{\sqrt{2n/45}}\to 𝓝\left(0,1\right) \end{array}$$ (4)

under the Yule or coalescent model, and

$$\begin{array}{c} \frac{C_{n}-n/4}{\sqrt{n/16}}\to 𝓝\left(0,1\right) \end{array}$$ (5)

for a uniform tree [12].

These two metrics complement each other well, as the number of cherries reveals recent asymmetry in the tree, whereas Sackin’s index gives the asymmetry of the tree over the whole evolutionary history [5]. In addition, these metrics are only weakly correlated, unlike for example, the Sackin and Colless indices [16, 17].

### Local asymmetry

The ordering of nodes in a rooted tree means we can consider the asymmetry in the phylogenetic tree throughout the evolutionary period, and not just at the root. This asymmetry could be due to a small effect at each internal node accumulating throughout the tree, or due to one or more nodes with highly imbalanced subtrees below them. Calculating the asymmetry over the entire course of the tree allows us to identify local asymmetry, even when there may not be significant evidence for global asymmetry (as obtained by considering the cumulative statistics at the root of the tree).

**Fig 1:**
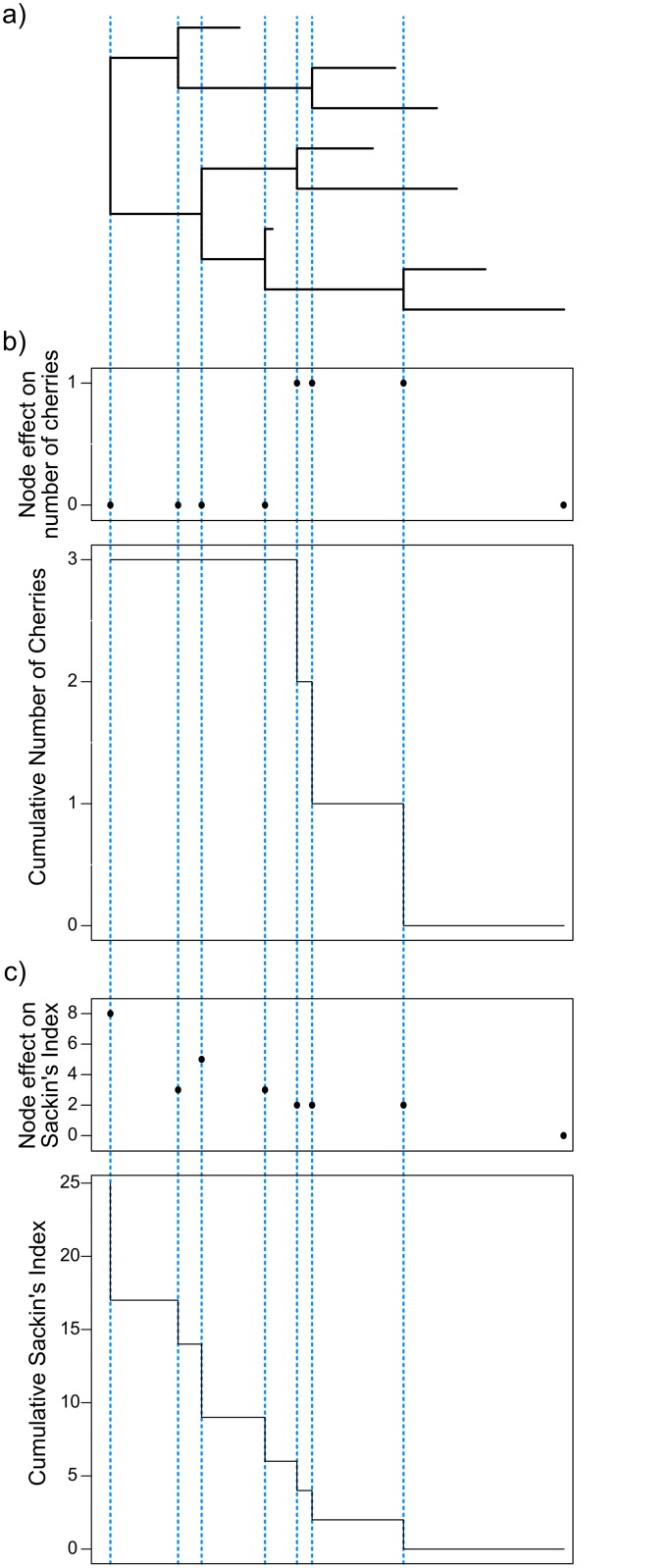
Calculating local asymmetry. For each internal node of an observed tree (a) it is possible to calculate the node contribution and cumulative number of cherries (b) and Sackin’s index (c).

There are two main types of event that can affect the shape of a phylogeny: a coalescence, and a new sampling event, which adds a tip. Sackin’s index and the number of cherries are both concerned with internal nodes rather than the tips, so we need only consider the former. At each coalescent event, we consider the contribution of that node to the overall metric. This results in a vector of *n* − 1 values, one for each ancestral node, giving a measure of how asymmetric the subtree below the node is (Fig 1). We can add these values cumulatively as we go backwards in time from the present towards the root, to investigate how asymmetry builds up over the course of the tree.

For the number of cherries, calculating the effect of each individual node is straightforward—being 1 if the node is a cherry (i.e. the direct ancestor of two tips) and 0 if it is not. To calculate the Sackin’s index for each node, rather than count the topological distance to the root for each tip as the calculation of the Sackin’s index is usually presented, we instead consider the number of times each node is traversed going from the tip to the root. Namely, this is the number of tips found in the subtree below the node of interest.

### Permuting the tree

**Fig 2:**
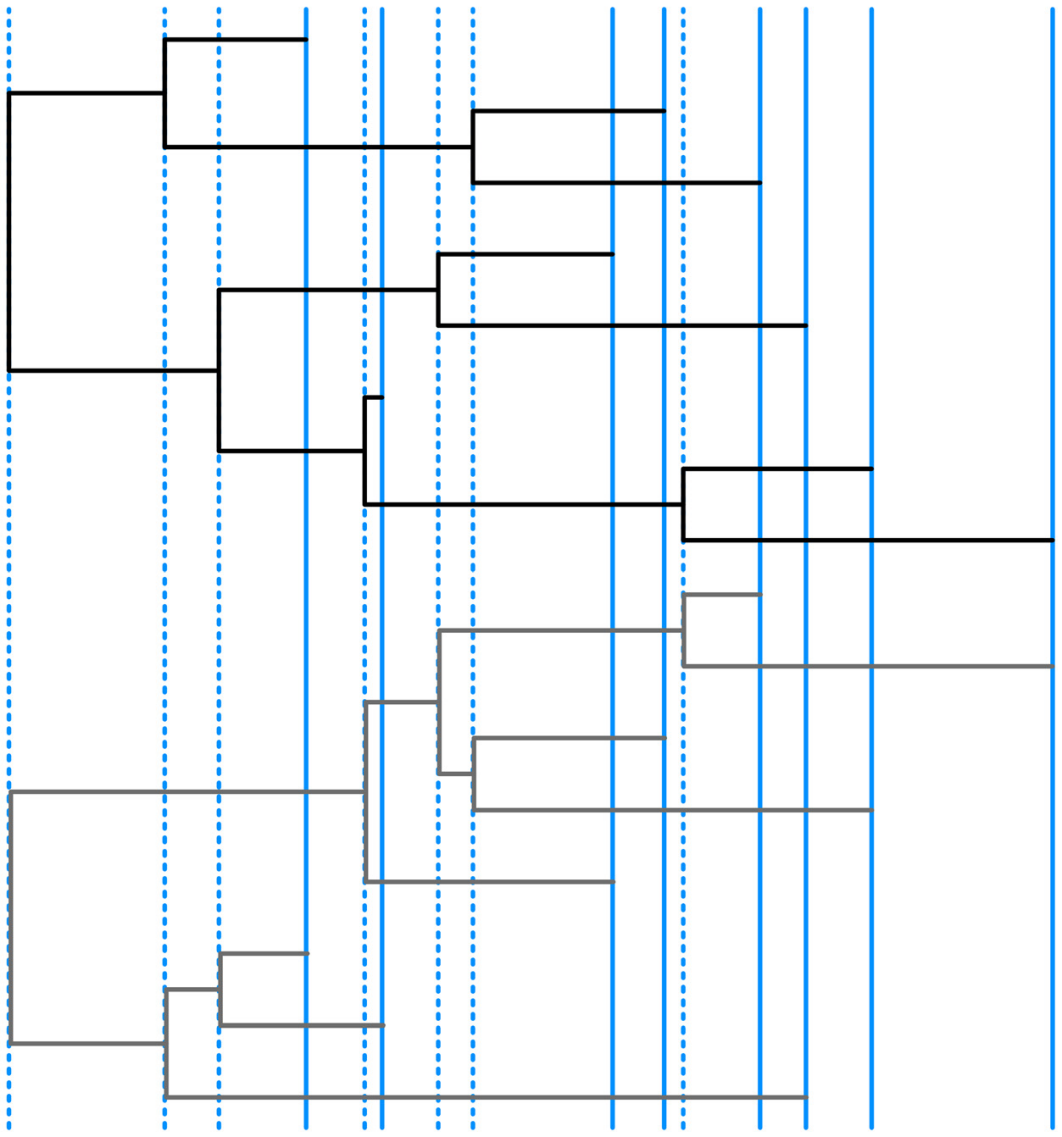
Permuting a time-stamped tree. The times of the tips (solid blue lines) and internal nodes (dashed blue lines) from the observed tree (top, black) are preserved in the permuted tree (bottom, dark grey).

To obtain the distribution of possible values for each the statistics for an observed tree, we permute the tree whilst retaining the same tip sampling and internal node times (Fig 2). These simulated trees form a neutrally evolving null distribution of coalescent trees, conditioned on the same tip and internal node times as the observed tree.

For *n* tips, there are *n* − 1 internal nodes. Starting at the time of the most recent internal node (say, *t*_1_) and going backwards in time from the present at *t* = 0, we consider all tips that were sampled more recently (i.e. between *t*_1_ and *t* = 0). Two of these tips are then chosen at random to coalesce, thus creating the internal node for *t*_1_. This continues backwards in time for each node in turn, with the only difference that coalescences can be between sample tips and nodes that have already been produced via a coalescence between the time to node *i*, *t*_*i*_, and the present.

The code to simulate permutations of an observed tree with the same sampling and coalescence times, and all the imbalance metrics considered above, were written in R [15], and are available as part of the treeImbalance package on GitHub (https://github.com/bdearlove/treeImbalance), and are in the Dryad Digital Repository: http://dx.doi.org/10.5061/dryad.v7817.

### Hypothesis testing

To obtain a distribution of possible values for each imbalance metric, 10,000 permutations of the observed tree with the same tip sampling and internal node times were generated. For each of these permuted trees, the number of cherries and Sackin’s index were calculated at each internal node and globally by computing the cumulative statistics at the root (Fig 1).

The median trajectory of Sackin’s index and the number of cherries throughout the ancestry of the tree (shown with a solid red line in plots) was calculated by partitioning around the medoid with a single cluster using the function pam in the cluster R package [18]. The medoid represents the trajectory which has the least dissimilarity with all the other possible trajectories from the permutation test. This ensures that the median is obtained from within the set of permuted trajectories, thus ensuring it is a ‘viable’ trajectory, and overcomes issues associated with other methods (such as calculating the mean or median statistic at each node), which do not necessarily force the trajectory to be monotonically increasing.

At each internal node, the 95% confidence interval was calculated by inverting the hypothesis test around the medoid value at that timepoint [19]. The medoid was subtracted from the permuted trees, and then the critical points of this distribution are found where 2.5% of the values are as or more extreme (with no interpolation). The confidence interval then is obtained by adding these back to the medoid. Calculating the 95% confidence in this way, as opposed to using quantiles or the variance, ensures that the value calculated is within the permuted dataset. Since considering the local imbalance at each node results in a multiple hypothesis test, several p-value adjustments were considered in order to control the family-wise error rate (including the Bonferroni correction and methods proposed by Holm (1979), Hochberg (1988) and Hommel (1988) [20–22]), and the false discovery rate (including methods proposed by Benjamini and Hochberg (1995) and Benjamini and Yekutieli (2001) [23, 24]). For the latter, we also investigated the q-value, which estimates the proportion of significant hypotheses that are false [25–27]. Results were generally consistent (S1 Table), so here we report the most conservative adjustment, the Bonferroni correction, alongside the unadjusted p-values. The uncorrected values remain valuable since the purpose of the test is to identify potential deviations from the model for further investigation, rather than necessarily a strict hypothesis test.

For the cumulative statistics, the Bonferroni correction is equal to the number of internal nodes, *n* − 1. For the single node contribution to Sackin’s index, the correction is *n* − 2, since at the root *n* tips will always be added.

### Simulated trees

To illustrate the bias of standard metrics, we simulated two sets of trees—one set with homochronous sampling (tips sampled at the same time) and one set with heterochronous sampling (tips sampled at different times). These were generated using Serial SimCoal [28] under a coalescent model with effective population size of 10^4^, with 100 tips sampled in the present for the homochronous sampling, and sampled over 10 time points each 1000 generations apart for the heterochronous sampling.

### Phylogenies

A single tip-dated phylogeny is required as input for our permutation approach. These can be obtained via a number of methods, but for viral datasets, the use of BEAST [29] is most common. Before implementing the permutation test, the observed trees were checked for polytomies, which were subsequently resolved into randomly ordered dichotomies with zero branch lengths. Negative branches were set equal to zero.

Tree files were available for the ebola virus [30] and influenza A virus [31] datasets in Newick format, and are available in the Dryad Digital Repository: http://dx.doi.org/10.5061/dryad.v7817. For the within-host HIV dataset [32], the sequences were aligned using MUSCLE v3.8.31 [33] and the maximum clade credibility tree (MCC) obtained using BEAST 1.8 with a GMRF Bayesian Skyride coalescent model [29]. The GTR model of nucleotide substitution [34] was used with an uncorrelated log-normal relaxed clock and a discretised gamma distribution with four categories was used to model rate heterogeneity across the sequence [35]. For the log-normal relaxed clock parameters, a uniform prior between 0.0 and 1.0 × 10^100^ was assumed for the mean, and an exponential with mean 1/3 for the standard deviation. A uniform (Dirichlet) prior was used for the nucleotide frequencies. The MCMC was run for 1 billion iterations, with a 10% burn-in period and samples saved every 10,000 iterations.

The within-host HIV skyride plot was obtained from the observed tree in R using an approximate approach that employs an integrated nested Laplace approximation [36].

## Results

In this section, we apply our test for detecting asymmetry in phylogenies with tips sampled at different times. The permutation test simulates new coalescent trees, conditional on the internal node and sampling times in the observed tree, so that a null distribution of asymmetry statistics can be calculated. The observed Sackin’s index and number of cherries can then be compared with this distribution to assess how asymmetric the observed tree is, compared to trees with those times.

**Fig 3:**
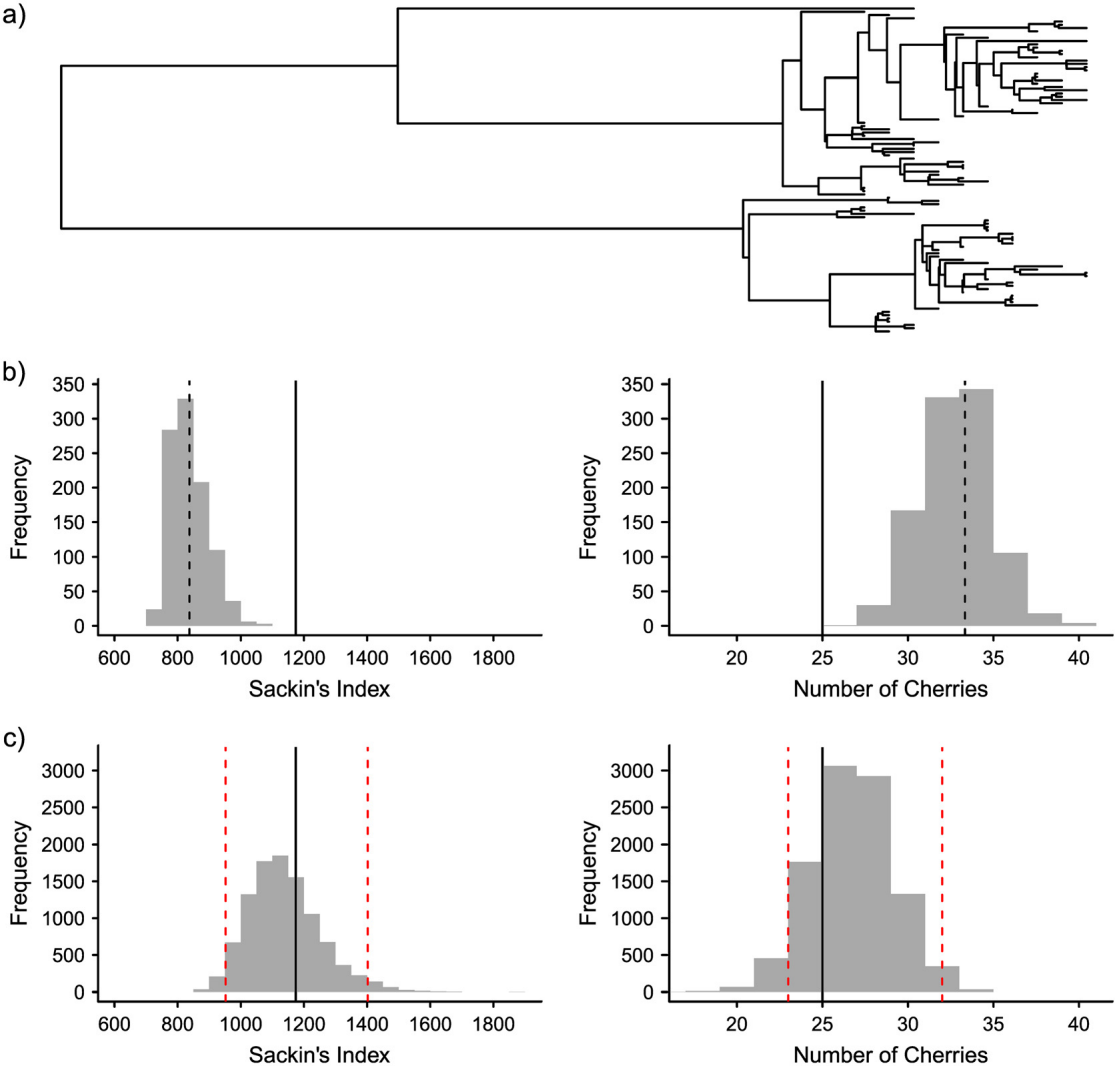
Permutations of an observed tree can overcome bias in detecting asymmetry in time-sampled phylogenies. a) An ‘observed’ tree, simulated under the coalescent model with 100 sequences sampled over 10 time points, each 1000 generations apart, with effective population size of 10^4^. b) The distribution of Sackin’s index and number of cherries for 100 random trees, simulated as in a) except for tips being sampled at a single time point. Expected values for these distributions are shown with dashed black lines. The observed values (solid black line) are highly extreme due to the implicit bias caused by tips sampled early in the ancestry. However, this is not the case when comparing them to a distribution calculated from permuting the observed tree, as seen in c), where there is no evidence to suggest the observed tree is asymmetric and the solid black line falls between the 2.5% and 97.5% quantiles (dashed red lines).

As an example, consider a tree simulated with 100 tips sampled over 10 time points (Fig 3a). Comparing this heterochronous tree with 1000 similarly simulated trees but with tips sampled at a single time point (Fig 3b) illustrates how extreme the observed values of Sackin’s index and number of cherries (solid black line) are compared to the expected values (dashed black line), purely due to the serial sampling [5]. However, when the heterochronous observed tree is compared to a distribution obtained from the permuted trees (Fig 3c), it can be seen that in the distribution of possible trees with the same internal node and tip sampling times, there is little evidence to suggest that this observed tree is asymmetric.

We tested this pattern for a total of 100 similarly simulated trees with heterochronous tip sampling. Using the standard metrics, 99 trees were found to be more asymmetric than expected using Sackin’s index, and 74 using the number of cherries. In contrast, using the permutation test with 10,000 simulated trees to control for the temporal signal, only two trees were significantly more asymmetric than expected with Sackin’s index when compared to the 2.5% and 97.5% quantiles, and only one tree using the number of cherries.

### Influenza A H5N1

We considered 98 influenza A virus H5N1 haemagglutinin sequences sampled from various bird species around seven locations in Asia (as distributed with BEAST v1.8.0, data originally collated by Wallace et al. (2007) [29, 31]). Here, we would reasonably expect that there could be three main sources of asymmetry in the phylogeny: the temporal sampling, selection and population substructure in the form of host species and location. Using the standard Sackin’s index, the phylogeny is found to be extremely asymmetric (p-value <0.0001), though there is not enough evidence to reject the null hypothesis of asymmetry at the tips using the number of cherries (p-value = 0.100).

**Fig 4:**
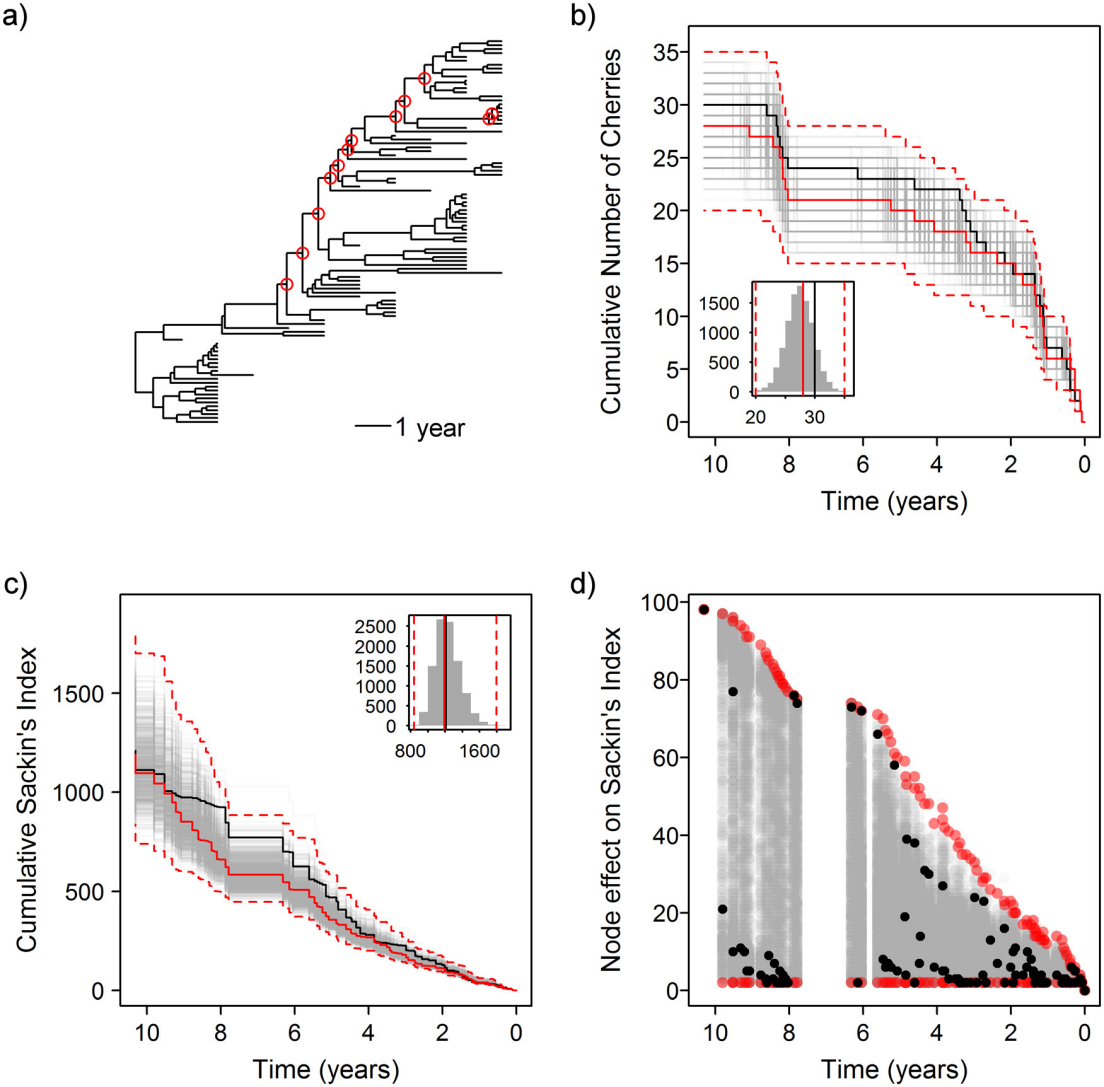
Asymmetry in influenza A H5N1. a) Tree of 98 influenza A H5N1 haemagglutinin sequences sampled from bird species in Asia. b) Observed cumulative number of cherries over time (black), with results from permuted trees (grey). Inset histogram shows global results. Red lines show the medoid (solid) and 95% confidence interval of the permuted results (dashed). c) Trajectories for the cumulative Sackin’s index. d) Node effect on Sackin’s index over time. Nodes which are significant at an unadjusted p-value of 5% are shown by an open red circle.

However, when we condition on the heterochronous sampling and coalescence times using the permutation test, we find that there is no evidence for global asymmetry with either statistic (Fig 4). There may be evidence of individual nodes being more asymmetric than expected, with 12 nodes significant at an unadjusted significance level of 5%, though none remain significant after the Bonferroni correction (Fig 4a). This suggests that the extreme result seen with the standard Sackin’s index was due to non-epidemiological effects, rather than heterogeneity in the population. However, the unadjusted p-values may still hint towards a deviation from the model so it could be worth investigating a model that allows for heterogeneity.

### Ebola virus

The 2014 West Africa epidemic of ebola virus is the largest known outbreak of the virus, causing 25,791 cases and 10,689 deaths (as of 15th April 2015) across Guinea, Liberia and Sierra Leone [37]. A recent study by Gire et al. [30] investigated sequences from 78 patients in Sierra Leone, suggesting a central African source to the outbreak in 2004 with continued human-to-human transmission, as opposed to punctuated re-transmission from a zoonotic source. A subsequent paper by Volz and Kosakovsky Pond [38] found strong evidence for superspreading, with much variance in the number of onward transmissions per individual, in contrast to the results of Stadler et al. who found that using two classes of transmission rates did not offer a significant improvement over an unstructured model [39]. Volz and Kosakovsky Pond note that this heterogeneity in transmission causes highly imbalanced phylogenies. Using a different method, Łuksza, Bedford and Lässig, identified a clade with a significantly higher growth rate than the ancestral clade it diverged from—again providing evidence for deviation from a simple randomly mixing model [40].

**Fig 5:**
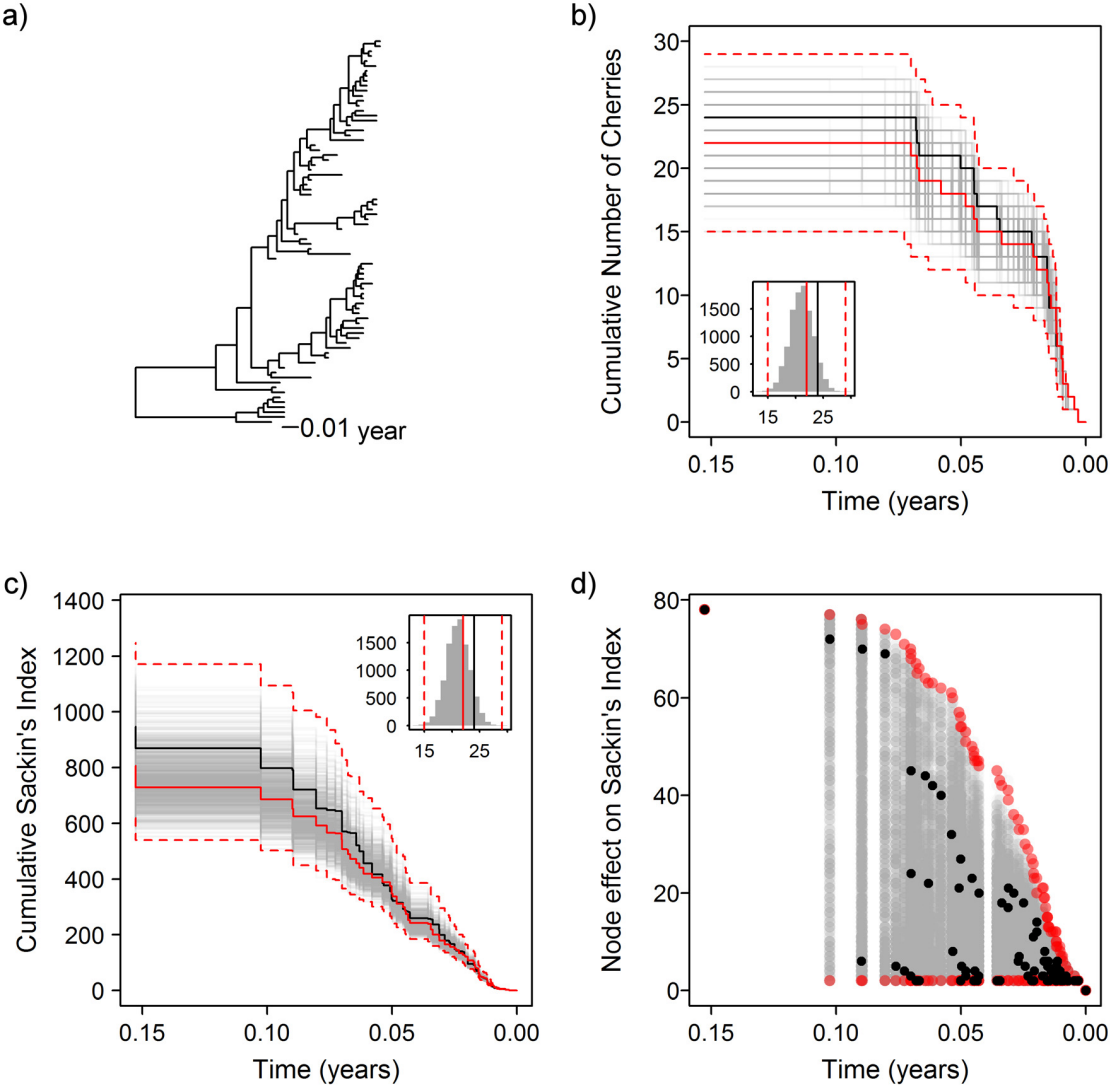
Asymmetry in the Sierra Leone ebola epidemic. a) Tree of 78 ebola virus whole genome sequences. b) Observed cumulative number of cherries over time (black), with results from permuted trees (grey). Inset histogram shows global results. Red lines show the medoid (solid) and 95% confidence interval of the permuted results (dashed). c) Trajectories for the cumulative Sackin’s index. d) Node effect on Sackin’s index over time.

Similarly to the influenza data, the standard Sackin’s index showed evidence of global asymmetry (p-value <0.0001), whilst the null hypothesis could not be rejected for the number of cherries (p-value = 0.141). Fig 5 shows the trajectory plots for the same statistics using the permutation test, showing that when controlling for the tip sampling being heterochronous, there is no evidence for asymmetry. Again, this suggests that the extreme result was due to non-epidemiological effects rather than heterogeneity in the tree. This clearly does not fit with what previous work has revealed about the dynamics of the epidemic, and it may reflect the limited power of these statistics compared to models that take the full phylogeny into account.

### Within-host HIV

**Fig 6:**
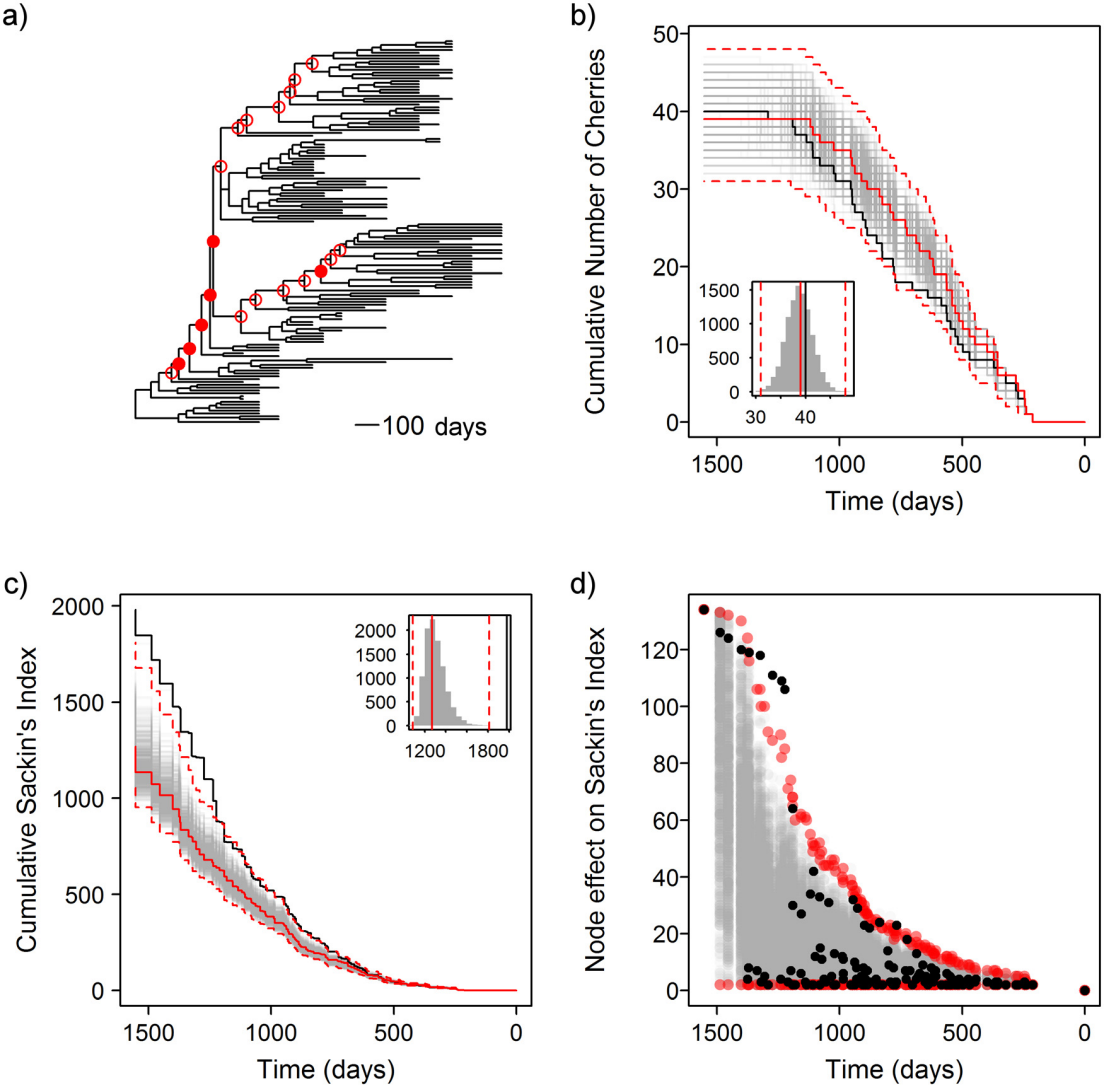
Within-host asymmetry is not always due to immune selection. a) Tree of 134 HIV envelope sequences from patient 83 [32]. b) Observed cumulative number of cherries over time (black), with results from permuted trees (grey). Inset histogram shows global results. Red lines show the medoid (solid) and 95% confidence interval of the permuted results (dashed). c) Trajectories for the cumulative Sackin’s index. d) Node effect on Sackin’s index over time. Nodes identified as significantly more asymmetric than expected with the Bonferroni correction are marked with a filled red circle in a), and those which are significant at an unadjusted p-value of 5% are shown by an open red circle.

Within a single host infected with HIV, we might expect that selection driven by neutralising antibodies would be the primary driver of asymmetry in the phylogenetic tree of the viral envelope, as rates of diversifying selection are significantly higher in HIV-1 *env* in individuals with robust neutralising antibody responses [32]. However, this is not the only cause of asymmetry in a phylogeny. We re-examined the HIV *env* sequence data of a patient who was previously shown to have a slow rate of immune escape from neutralising antibodies [32]. There were 134 full-length *env* sequences available, collected from 13 time points sampled over 1,098 days of follow up (Fig 6a). This phylogeny was found to be asymmetric with the standard Sackin’s index (p <0.0001), and was also significant using the number of cherries (p = 0.028).

Correcting for the tip sampling with the permutation test, the number of cherries shows no evidence of global asymmetry in the phylogeny, though suggests there is some evidence of recent local asymmetry between 767 and 781 days from the present (Fig 6b). Sackin’s index shows strong global asymmetry at the the root, which accumulates throughout the depth of the tree (Fig 6c). Within this, there are six individual nodes identified as having more asymmetric than expected subtrees below them (Fig 6d) with the Bonferroni correction, and 20 at the unadjusted 5% level. If we consider the q-value instead, there are 14 nodes with a q-value of 2.5% in the upper tail (for a 5% two-tailed test), suggesting that less than one of them (0.35) will be a false negative.

Examining the skyline plot for these data (S1 Fig) does not indicate any deviations from the null model. There are two distinct clades circulating within the patient at the same time in the tree, and if these clades were non-overlapping in time, we would see a pronounced dip in the skyline plot. This is not the case, with the effective population size instead showing steady exponential growth. This pattern and treeshape is reminiscent of the inter-subtype competition identified by Ferguson, Galvani and Bush [41].

## Discussion

In this paper, we have presented a framework to quantify asymmetry in phylogenetic trees where the tips have been sampled at different times. Previously, it has been highlighted that understanding the link between a tree topology and the evolutionary processes that gave rise to it is difficult [6, 42], which is further confounded by the fact that standard tests for asymmetry are implicitly biased in trees with heterochronous sampling [5]. The permutation test described here allows an observed phylogenetic tree to be compared to a distribution of coalescence trees, conditional on the same internal node and tip sampling structure. This is in contrast to the Temporal Clustering (TC) statistic proposed by Gray et al. [43], which tests for a ‘temporal signal’ in a tip-dated phylogeny, whereby sequences sampled around the same time are found clustered together in the tree and among these is the ancestor of any clade with sampling dates closer to the present. Their statistic permutes the tips with a fixed tree, whereas the test presented here permutes the tree conditional on the observed temporal structure in the form of tip sampling dates and internal node times. Trees with high temporal clustering have a higher potential for false positives from the standard global tests. The three datasets presented in this paper all have a strong temporal signal according to their TC statistic. However, when we control for their temporal structures, they display different levels of asymmetry.

Although only Sackin’s index and the number of cherries were illustrated here, the permutation test can be extended to other metrics of asymmetry including Colless’ index [10], Shao and Sokal’s balance statistics *B*_1_ and *B*_2_ [44], and the shape statistics of Agapow and Purvis [45, 46]. These statistics use varying measures of topological distance to quantify asymmetry, meaning they tend to be biased when tips are sampled earlier in the tree and have fewer nodes connecting them to the root and other tips of the tree. Given that the number of cherries and Sackin’s index did not have the power to identify the asymmetry present in the ebola tree, it may well be worth considering a wider range of statistics alongside the permutation test if there is strong external suggestion of asymmetry in the tree. It is important to note that the branch lengths in a phylogeny can also convey important information about the dynamics of disease. The kernel function of Poon et al. [42] accounts for differences in branch lengths when comparing multiple trees, but cannot be used to statistically assess a single observed tree on its own. However, our permutation test could be used alongside this method to calculate the distance between the observed tree and simulated null trees. Additionally, the topology and the branch lengths of a viral phylogeny are not necessarily equivalent to the underlying transmission tree [47], and therefore it is important to be aware of the possible discrepancy in equating asymmetry in the phylogeny with asymmetry in transmission.

Generally, more complicated models will better fit the data. However, increased model complexity can be computationally intensive. As such, the model that is considered the best comes from a balance of the scientific relevance (the biological plausibility), the goodness of fit, and complexity [48]. While this usually relies on some simplifying assumptions, these are often violated—such as the assumption of a randomly mixing population. As a result, it is important to bear in mind the overall fit of the model to data. In the Bayesian framework, posterior predictive simulation is widely used for model checking, but despite recommendations for its use in the literature [49–53], it remains underutilised in the field of phylogenetics. In addition, these tests are often only possible alongside or once the analysis has been completed, after much computational effort. Since the base topology can often be recovered relatively quickly and accurately, our permutation test represents a quick method for checking whether the assumption of random mixing is supported, or whether there is evidence of asymmetry and therefore heterogeneity in the population.

We simply test for evidence of asymmetry in an observed tree, which can arise in the tree due to many processes in the underlying population such as contact rates and population structure [5, 7]. As evidenced with the within-host HIV data, it is not necessarily simple to interpret the underlying cause of local asymmetry being detected. It might be preferable to control for certain aspects of asymmetry occurring in the tree (that is, allow for some specific asymmetry in the null model), and see if there is significant evidence for further imbalance beyond that expected under the null model. However, methods that have become standard for inferring structure in the phylogenetic tree, such as the phylogeographic approach of Lemey et al. [54], make the assumption that the tree branching structure is not affected by the heterogeneity in the population (i.e. the population is randomly mixing, and the discrete trait model is simply overlaid over the tree). Thus, our permutation test can be used to justify whether this is an appropriate assumption, or whether it might be more advisable to use a more complex model such as the structured coalescent [55, 56].

Our approach is fast, has a free software implementation, and can offer important additional insights by highlighting potential lack of goodness-of-fit of commonly used coalescent and birth-death models.

## Supporting Information

S1 Fig Skyride ride plot for the within-host HIV phylogeny showing exponential growth. Dashed lines show the 95% confidence interval, and red vertical lines indicate the timing of nodes evidence of higher than expected asymmetry in the tree.
(TIFF)

S1 Table Coverage probabilities for the permutation tests for the cumulative Sackin’s index (a), node effect on Sackin’s index (b) and cumulative number of cherries (c). Within datasets, for each permuted tree from the null distribution the probability of seeing a statistic as or more asymmetric in the remaining 9,999 permuted trees was calculated. The number of significant nodes was recorded, with the coverage probability being the proportion of trees for which there were no nodes with evidence of asymmetry. For a two-tailed hypothesis test with 95% confidence, we would expect the coverage to be around 97.5% showing that the unadjusted p-values give more false positives than expected, and that all adjustments should be conservative.
(PDF)
