# Supplementary figures and images for: Measuring Asymmetry in Time-Stamped Phylogenies
Source: PLoS Comput Biol. 2015 Jul 6;11(7):e1004312. doi: 10.1371/journal.pcbi.1004312 (PMC4492995; doi:10.1371/journal.pcbi.1004312)

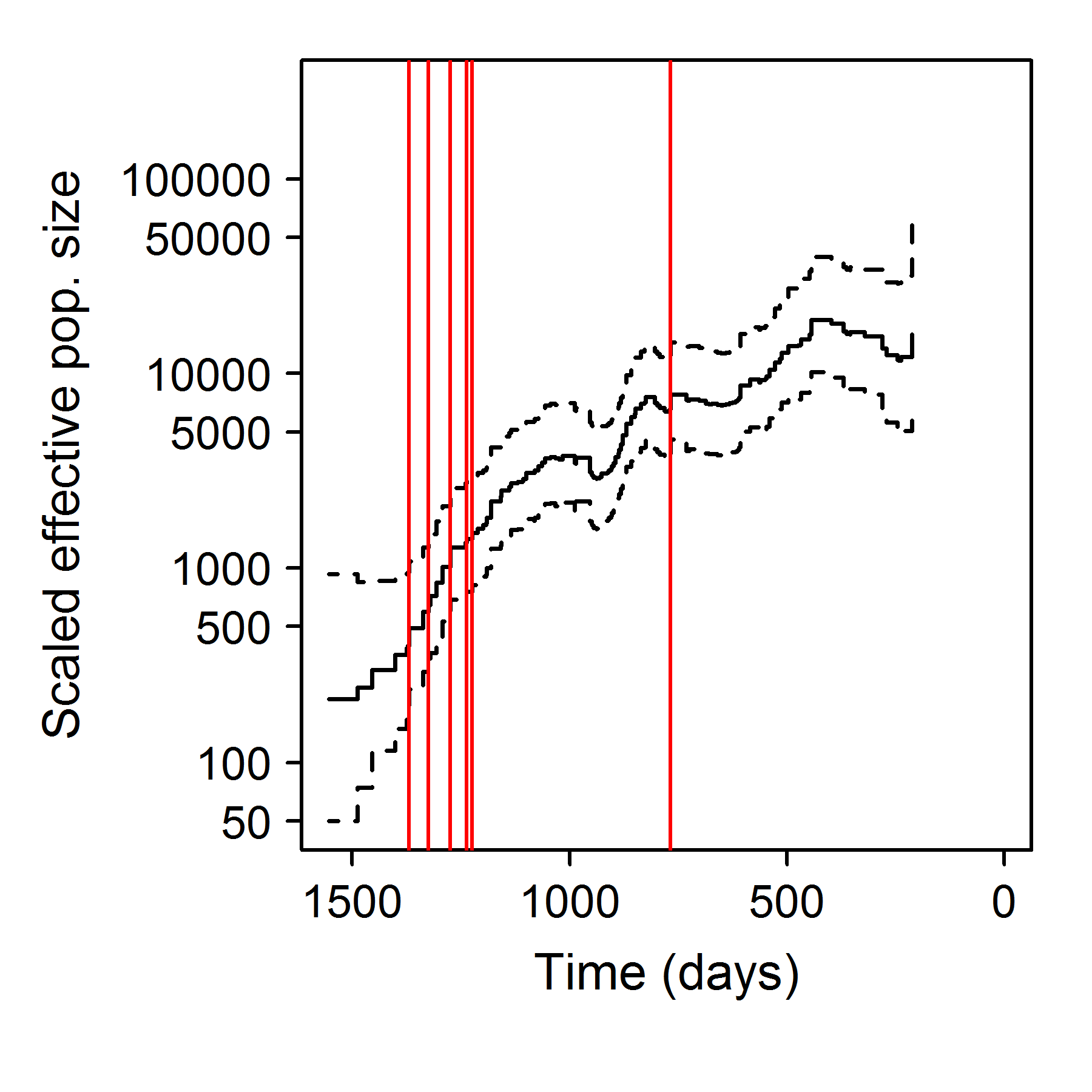

Supplement: S1 Fig — Dashed lines show the 95% confidence interval, and red vertical lines indicate the timing of nodes evidence of higher than expected asymmetry in the tree. (TIFF) [file pcbi.1004312.s001.tiff]
