# Supplementary material for: Measuring Asymmetry in Time-Stamped Phylogenies
Source: PLoS Comput Biol. 2015 Jul 6;11(7):e1004312. doi: 10.1371/journal.pcbi.1004312 (PMC4492995; doi:10.1371/journal.pcbi.1004312)
Supplement: S1 Table — Within datasets, for each permuted tree from the null distribution the probability of seeing a statistic as or more asymmetric in the remaining 9,999 permuted trees was calculated. The number of significant nodes was recorded, with the coverage probability being the proportion of trees for which there were no nodes with evidence of asymmetry. For a two-tailed hypothesis test with 95% confidence, we would expect the coverage to be around 97.5% showing that the unadjusted p-values give more false positives than expected, and that all adjustments should be conservative. (PDF) [file pcbi.1004312.s002.pdf]

a) Coverage for the cumulative Sackin's index.

Adjustment	Within-host HIV	Influenza H5N1	Ebola
None	0.7132	0.7071	0.7358
Holm (1979)	0.9950	0.9925	0.9925
Hochberg (1988)	0.9950	0.9925	0.9925
Hommel (1988)	0.9945	0.9924	0.9925
Bonferroni	0.9950	0.9925	0.9925
Benjamini and Hochberg (1995)	0.9892	0.9889	0.9891
Benjamini and Yekutieli (2001)	0.9960	0.9969	0.9971

b) Coverage for the node effect on Sackin's index.

Adjustment	Within-host HIV	Influenza H5N1	Ebola
None	0.2485	0.3890	0.4544
Holm (1979)	0.9863	0.9876	0.9892
Hochberg (1988)	0.9863	0.9876	0.9892
Hommel (1988)	0.9863	0.9876	0.9892
Bonferroni	0.9863	0.9876	0.9892
Benjamini and Hochberg (1995)	0.9842	0.9858	0.9879
Benjamini and Yekutieli (2001)	0.9935	0.9954	0.9970

c) Coverage for the cumulative number of cherries.

Adjustment	Within-host HIV	Influenza H5N1	Ebola
None	0.8528	0.8635	0.8748
Holm (1979)	0.9983	0.9979	0.9973
Hochberg (1988)	0.9983	0.9979	0.9973
Hommel (1988)	0.9980	0.9979	0.9972
Bonferroni	0.9983	0.9979	0.9973
Benjamini and Hochberg (1995)	0.9945	0.9953	0.9946
Benjamini and Yekutieli (2001)	0.9987	0.9989	0.9988
